# Novel insights into biomarkers of progression in Desmoid tumor

**DOI:** 10.3389/fonc.2023.1206800

**Published:** 2023-08-03

**Authors:** Baiqi Liu, Zefang Sun, Rui Zhou, Dingcheng Shen, Shuai Zhu, Lu Chen, Gengwen Huang

**Affiliations:** ^1^ Department of Hernia and Abdominal Wall Surgery, General Surgery, Xiangya Hospital, Central South University, Changsha, China; ^2^ National Clinical Research Center for Geriatric Disorders, Xiangya Hospital, Central South University, Changsha, China

**Keywords:** Desmoid tumor, predictive markers, progression, recurrence, active surveillance

## Abstract

Desmoid tumor (DT) is a rare neoplasm characterized by the proliferation of myofibroblastic cells that infiltrates and invades adjacent tissues. Due to its locally aggressive and recurrent nature, DT often causes local symptoms and can be challenging to manage clinically. Therefore, identifying biomarkers that can predict the progression of DT and guide treatment decisions is critical. This review summarizes several biomarkers that have been implicated in active surveillance (AS) and the prediction of postoperative recurrence and attempts to elucidate their underlying mechanisms. Some of these novel markers could provide prognostic value for clinicians, and ultimately help facilitate optimal and accurate therapeutic decisions for DT.

## 1 Introduction

Desmoid tumor (DT), also known as aggressive fibromatosis (AF), is a rare and locally invasive soft tissue tumor, which occurs in approximately 3-5 individuals per million per year (1). It is estimated that 85% of DT cases are sporadic, while 3.5-32% of cases are related to familial adenomatous polyposis (FAP) or Gardner’s variant (2).

DT is characterized with monoclonal myofibroblast proliferation, which originates from musculoaponeurotic structures and may occur in the abdominal, chest walls, mesenteric root and extremities (3). Previous trauma history, genetic factors and pregnancy are all closely related to the etiology of DT (3).

Despite 20-30% of spontaneous regression or resolution in DT, the high recurrence rate(25-77%) of DT poses a long-term treatment dilemma (3, 4). DT patients face substantial challenges due to the unpredictable course and the uncertainty of treatment effect. While surgical resection was once the preferred treatment, it is now considered invasive, complex, and prone to recurrence. Even with negative resection margins, the recurrence rate was as high as 39.3% (5). Surgical resection with a safety margin is recommended only when tumor is large and causing significant symptoms. Currently, active surveillance (AS) is recommended based on results from numerous clinical trials and observational studies (3). AS and surgical treatments did not differ significantly in RFS over three years (6). If patients with DT have minimal or no symptoms, 2-3 monthly clinical and radiographic observation is recommended. Nevertheless, the unpredictable natural history of DT and lack of monitoring biomarkers make clinical decisions difficult during AS. Therefore, it is essential to discover stable biomarkers that can be used to predict progression and guide treatment direction. In the current review, we summarized a series of novel biomarkers with the aim of providing options for monitoring the clinical progression and recurrence of DT.

## 2 Pathway

### 2.1 Wnt pathway

**Figure 1:**
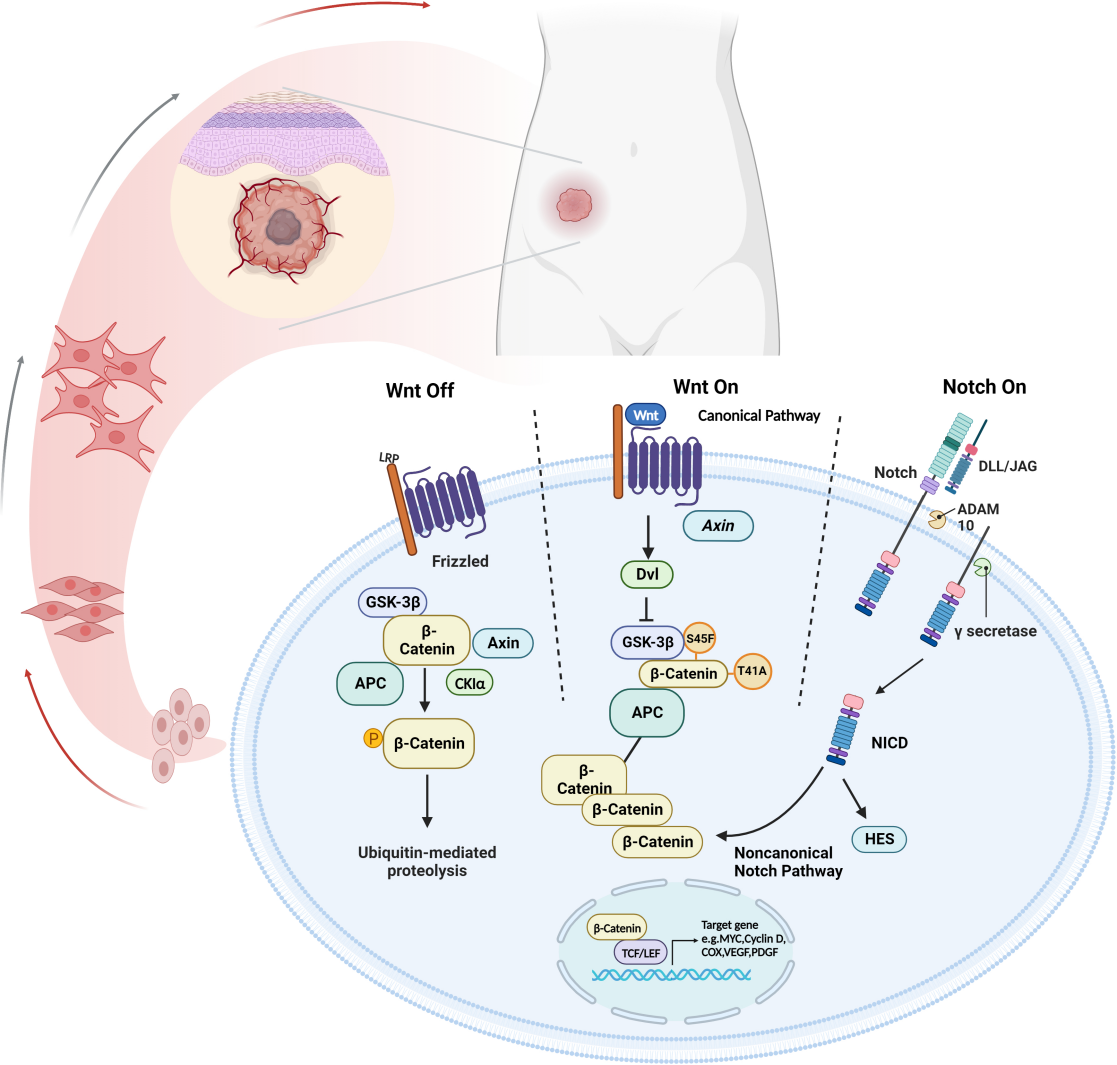
The cross-talk between Wnt/β-catenin and Notch signaling pathway. The canonical Wnt signaling pathway suppresses β-catenin ubiquitination to stabilize β-catenin expression, allowing it to contact TCF/LEF to upregulate pro-tumor factors. Notch signaling results in the progression disease progression through NICD in a non-canonical Notch pathway. *Created with Biorender.com*.

Wnt pathway is an evolutionarily conserved signaling pathway that plays a key role in organ development and the function of various tissues (7). When the Wnt is inactivated, the complex consisting of GSK-3β, APC, CK1α and Axin performs sequential phosphorylation reactions at β-catenin. The phosphorylation of the β-catenin results in the ubiquitination and subsequent degradation by β-TrCP and its proteasome, which in turn increases the β-catenin steady-state level (8, 9). While binding with activated Wnt, Dvl and LRP5/6 phosphorylate and inactivate GSK-3β, preventing β-catenin from being phosphorylated and degraded. These processes cause β-catenin to accumulate in the nucleus, which binds with TCF/LEF to activate downstream target genes including *MYC*, *COX*, *Cyclin D*, *PDGF*, *VEGF*, etc. (8, 9) (**Figure 1**).

The activated Wnt pathway is closely associated with tumorigenesis (10–12). Numerous studies have shown that the Wnt signaling components, including β-catenin and APC, are essential in DT. Approximately 85-90% of sporadic DT accompanies with the mutation of *CTNNB1*, and most of the remaining cases are related to *APC* mutations (3). COX, VEGF and Cyclins are shown as representative downstream signaling molecules, contributing to the progression of DT. They are implicated in several pathophysiological mechanisms, including cell proliferation, invasion, angiogenesis and apoptosis (13). Thus, the molecules involved in the Wnt pathway may provide valuable prognostic targets for DT.

### 2.2 Notch pathway

A series of studies have shown that the Notch signaling pathway participated in cancer development by regulating cell proliferation, apoptosis, and differentiation (14–16). A canonical Notch signaling consists of Notch 1–4, DLL-1/3/4, JAG1/2 and CBF-1. Initiated by either ADAM10 or ADAM17, Ligand-activated Notch receptors undergo multiple proteolytic cleavages, forming the transmembrane fragment Notch. The proteolytic product transforms into the NICD after a second proteolytic cleavage, which is controlled by γ-secretase. NICDs enter the nucleus and interact directly with the CSL complex, regulating the expression of downstream genes, such as HEY and HES (14, 17).

The Notch-related molecules, including HES and ADAM, can be used as clinical markers for the diagnosis of DT. These molecules have been used to further distinguish DT from hypertrophic scars (18, 19). As for the treatment of advanced and progressive DT, preliminary data from the clinical trial showed 71% of advanced DT patients partially responded to oral γ-secretase inhibitors (GSI) PF-03084014 (20). Additionally, several clinical trials(NCT01981551, NCT00878189) observed positive results from advanced and recurrent DT treated with GSI (21–23). These results indicated that some key elements in the Notch pathway might contribute to the progression and recurrence of DT (24).

Previous studies have shown that the crosstalk between the Notch signaling pathway and the Wnt signaling pathway facilitated tumor progression (24, 25) (**Figure 1**). Peignon G et al. elucidated that Notch activation was an early event in Wnt-induced intestinal tumorigenesis, and maintained throughout downstream from the Wnt/β-catenin cascade (26). In patients with FAP, Notch signaling was activated by β-catenin-mediated upregulation of JAG1 (27). Among the complex Wnt and Notch crosstalk, many abnormally expressed specific molecules may be potential significant biomarkers.

### 2.3 Other potential pathways

In addition to the Wnt pathway and the Notch signaling pathway, other pathways have been shown to be involved in DT initiation and progression. The PI3K/Akt pathway has been considered as a therapeutic target for DT, as the tyrosine-kinase receptors (e.g., VEGFR and PDGFR) are detected in DT (28). Some tyrosine kinase inhibitors, including imatinib, sunitinib and sorafenib, are currently being tested in different phases of clinical trials for DT (29, 30). Expression of estrogen receptor-β (ERβ) in DT samples suggests the specific functions of estrogen signaling, dictating distinct therapeutic options for DT (31–33). However, the individual response to anti-estrogen agents varies and evidence from prospective studies is limited. Besides the above, recent studies have indicated that several signaling pathways, including TGF-β signaling pathway, JAK/STAT signaling pathway and Hedgehog signaling pathway, might also be involved in the transformation and progression of DT (34–36).

## 3 Biomarkers in AS

### 3.1 Tumor tissue

#### 3.1.1 *CTNNB1* S45F mutation

*CTNNB1* mutations are common in DT patients. It is estimated that two types of *CTNNB1* mutations, T41 and S45F, account for 35% and 55% of DT patients respectively. Other types of mutations are relatively infrequent, including S45P, D32G, T41A, S45C, T42_K49delinsQ and H36del (4, 37, 38). Accumulating evidence indicates that the S45F mutation is associated with poor prognosis in DT patients (39–42) (**Table 1**). Several studies reveal that S45F mutation is more likely to occur in the extremities, and the prognosis of these cases is worse than other sites (37, 43). A recent prospective study has shown that S45F mutation is associated with tumor progression (HR = 6.24 [95% CI 1.92–20.30]) and suggests the onset of active treatment (AT) during the AS (44). Compared with tumor diameter, gender, recurrence cases and other clinical factors, S45F mutation is significantly related to 3-year RFS (45). It has been reported that *CTNNB1* mutation types was associated with tumor progression and aggressive treatment with adjuvant radiotherapy was administered accordingly. Follow-up showed no recurrence over 38 months, highlighting the value of CTNNB1 mutation type for guiding treatment strategies in DT (46). These findings suggested that S45F might be the most significant prognostic factor during the monitoring period. Further prospective studies with large sample sizes will give more solid evidence to guide clinical usage, especially for predicting recurrence and active surveillance.

**Table 1:** Biomarkers of progression in desmoid tumor.

Biomarker	Source	Change	Biological effect	Related clinical research		
				Author	Main outcome	Significance
Biomarkers in Active Surveillance						
CTNNB1	Tumor	S45F Mutation	• β-catenin induced transcriptional expression of pro-tumor factors (41, 47) • Immune evasion (40, 48, 54)	Hamada et al (39)	**CR, PR, SD**(n) **S45F(+)** 0/20 **S45F(-)** 20/20 **PD** **S45F(+)** 4/13 **S45F(-)** 9/13	p = 0.017
				Sakai et al (45)	**HR** (Multivariate) **1.96**	p = 0.048
				Schut et al (44)	**HR** (Multivariate) 6.24	P<0.05
				Kaspere et al (108)	**PAR_6mo_** **S45F** 85% **WT** 43%	p = 0.05
				Lazar et al (43)	**HR** (Multivariate)	
					**S45F** 3.50	p=0.0036
					**S45P** 1.13	p=0.8064
					**T41A** 1.11	p=0.8499
				Crago et al (37)	**HR** (Multivariate) 1.59	p = 0.41
				Colombo et al (49)	**No significant association**	P=0.06
CfDNA	Blood	*CTNNB1* Mutation Increased	• Tumor microenvironment (54)	Macagno et al (54)	**Plasmatic cfDNA concentration (copies/mL)**: **P:**1439 (CI 95%:900–1958) **NP:** 528.7 (CI:95%: 166.7–875)	p=0.00026
miR-143-3p	Blood	Increased	• Tumor cell proliferation	Yamano et al. (57)	**Significant association**	P=0.001
Biomarkers in Prediction of Postoperative Recurrence						
CTNNB1	Tumor	Mutation	• β-catenin induced transcriptional expression of pro-tumor factors (41, 47) • Autophagy and inhibited apoptosis (66)	Domont et al. (61)	**5-year RFS** **Mutation** 49% **WT 73%**	P=0.02
		S45F Mutation		Colombo et al (40)	**5-year RFS** **S45F 45%,** **Other mutation 66%** **WT 91%** **HR** (Multivariate) 2.59	p = 0.001 p=0.05
				Mullen et al (109)	**5-year RFS** **S45F** 59.8%, **T41A** 54.9% **WT** 73.6%	p=0.434
Cyclin A	Tumor	Increased	• Tumor cell proliferation (70)	Santti et al (70)	**HR** (Univariate) 1.9	p=0.02
Cyclin D	Tumor	Increased	• Tumor cell proliferation (47, 73)	Santti et al (32)	**Correlation with Ki67** **r=**0.40	p = 0.001
					**HR**(Univariate) **Varied** according to the used cutoff	p>0.1
Cox	Tumor	Increased	• Angiogenesis	Signoroni et al (110)	**IHC:** n (%) 8/8 (100)	–
PDGF	Tumor	Increased	• Angiogenesis	Signoroni et al (110)	**IHC** n(%) 8/8 (100)	–
ERβ	Tumor	Increased	• Tumor cell growth, differentiation and reproduction (87–89)	Santti et al (32)	**HR** (Univariate) 2.6	p = 0.02
PARP-1	Tumor	Increased	• Apoptosis (94, 111)	Bräutigam et al (94)	Survival cutoff ΔCt = 15.487	p=0.03
CTC	Blood	Increased	• Unclear in DT	Braun et al (103)	**ICC**: n (%) 16/16 (100%)	–

CfDNA Cell Free DNA, Cox Cyclooxygenase, PDGF Platelet derived growth factor, ERβ Estrogen receptor beta, CTC circulating tumor cell, PARP-1 Poly (ADP-Ribose) Polymerase 1, CR complete response, PR partial response, SD stable disease, PFS progression free survival, HR Hazard Rate, PAR_6mo_ progression arrest rate after 6 months.

To account for the strong tendency of S45F mutation toward the progression, the function of CK1α in the Wnt/β-catenin pathway may be relevant. β-catenin ubiquitination begins with the phosphorylation in position 45 amino acid residue by CK1α. With the mutation of this specific residue, the ubiquitination process can not completely begin, leading to the imbalance of β-catenin (40). A study showed that the CBNNT1 S45F mutant allelic replication promoted the overexpression of β-catenin in DT. Correspondingly, the downstream factors, including COX, MYC, VEGF, and PDGF, show increased expression patterns, which are involved in tumor progression (41, 47).

**Figure 2:**
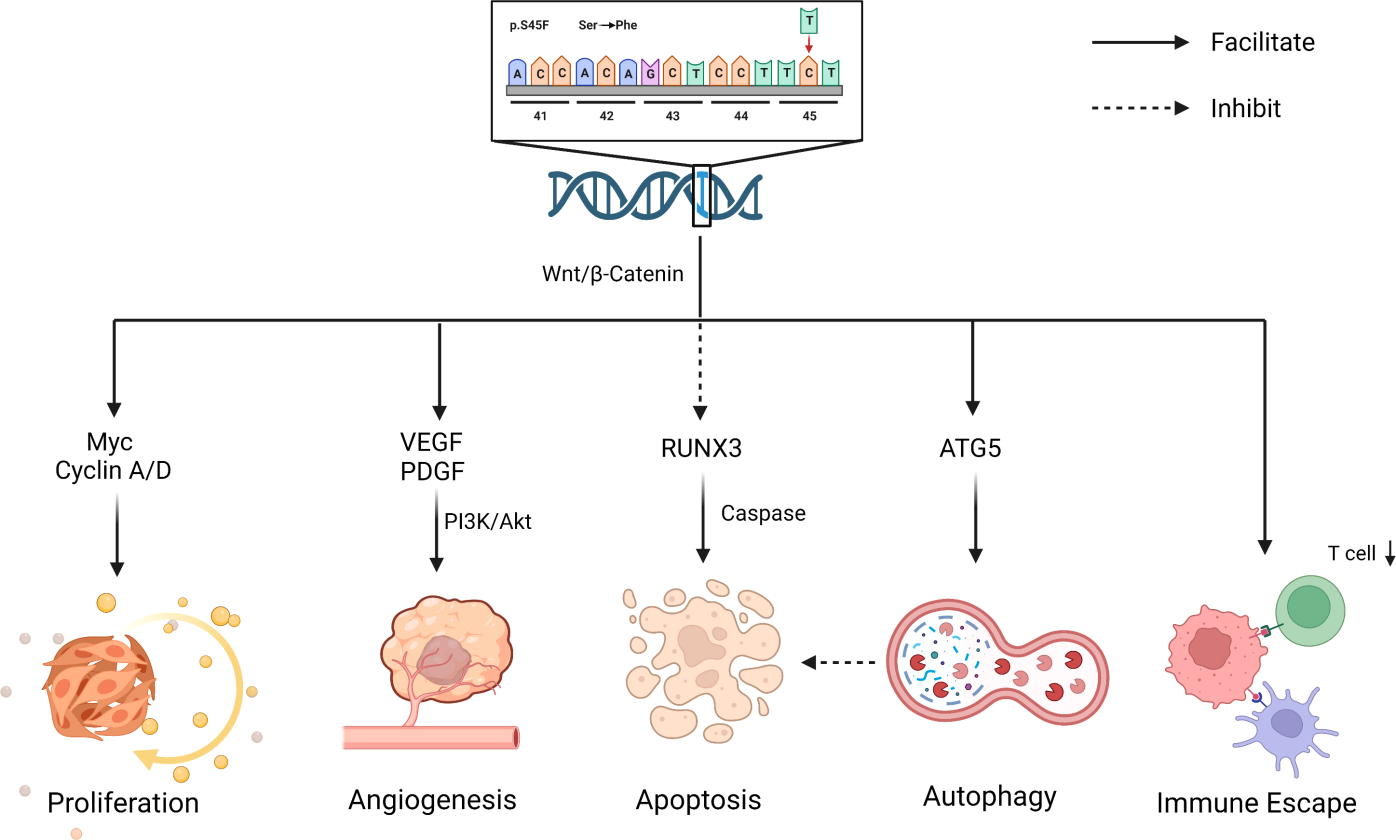
Biological effects for S45F mutation of *CTNNB1*. S45F mutation is involved in the progression and recurrence of DT, including autophagy, immune escape, proliferation, angiogenesis an inhibited apoptosis, etc. *Created with Biorender.com*.

In addition to affecting its own expression, the S45F mutation also works by influencing the immune response in the tumor microenvironment (TME). Colombo et al. observed that the two genetic lines (S45F/T41) exhibited different enrichment in immune-related genes (40). Inflammatory-defense-humoral immune response and antigen-binding related genes were enriched in T41F mutation cases, which corresponded with a high proportion of T cells at the tumor margin by immunohistochemical analysis. Instead, the mutation of S45F was found to be capable of increasing Treg cells and diminishing effector T-cell numbers, thereby promoting tumor progression by immune evasion (48). The discrepancy between S45F and other mutations may be due to the differences in the TME. However, the effect of TME on DT has not been clearly elaborated and deserves more exploration (**Figure 2**).

Although many studies support the association between CTNNB1 and the poor prognosis of DT, the relationship between *CTNNB1* mutation status and the clinicopathological characteristics of DT remains controversial. A follow-up prospective study detected that despite a trend toward the switch to AT, there is no significant association between RFS and the S45F mutation (49). Crago et al. found that “Wild types” defined by Sanger sequencing actually had *CTNNB1*, *APC* and other rare mutations(chromosome 6 loss and *BMI1* mutation) using next generation sequencing (37). Colombo et al. first detected two different large deletions of about 190 bp involving exon 3 of *CTNNB1* in two DT cases through separate analysis of unmapped reads and subsequent validation using PCR, which are difficult to be detected by conventional whole-exome sequencing (WES) analysis (50). These findings emphasize the challenges in detecting these deletions and a high level of tumor heterogeneity not previously described in DT. Because of these properties, larger samples and more precise sequencing methods are needed to confirm the clinical value of the *CTNNB1* mutation.

### 3.2 Peripheral blood

#### 3.2.1 Cell free DNA

CfDNA is a fragment of DNA released from tumor cells during necrosis or apoptosis. The cfDNA levels in the peripheral blood of most tumor patients are significantly higher than normal individuals (51). It has been demonstrated that cfDNA can be used to diagnose pancreatic cancer, colorectal cancer and other cancers (52, 53). Macagno et al. determined plasmatic cfDNA concentration and mutation from DT patients’ blood using a targeted Digital-droplet PCR (54). A significant correlation was found between the concentration of cfDNA and tumor progression (54). CfDNA level greater than 900 DNA copies/m provided 100% sensitivity and 76.5% specificity as the poor prognostic factor. When the cfDNA level was greater than 1375 DNA copies/m, the sensitivity was 57.14% and the specificity was 100%. Using these two thresholds(900 DNA copies/m and 1375 DNA copies/m), cfDNA could accurately predict the prognosis of DT in 65% of cases (54). However, it is noteworthy that there were inconsistent correlations between *CTNNB1* status in cfDNA and prognosis. This could be attributed to the fact that cfDNA is derived from the TME and the adjacent tissues rather than tumor cells. Based on the local invasive characterization, surrounding cells and inflammatory cells in the TME released wild-type cfDNA into the blood. CfDNA is more indicative of the invasion intensity of DT rather than tumor size (54). The finding indicates that cfDNA analysis may be clinically useful for DT patients, especially those who are under AS management.

#### 3.2.1 Circulating microRNA

MiRNAs are small non-coding RNA molecules that participate in RNA silencing and gene regulation post-transcriptionally, which are widely present in multiple diseases (55). There is growing evidence that circulating miRNAs can be used as a stable and reliable serological biomarker (56). Given the rarity of DT, the assessments of miRNAs on DT are limited. A recent study has investigated that the levels of circulating miR-143-3p were screened out as a candidate biomarker for FAP, compared with healthy controls (57). Notably, among these FAP patients, the miR-143-3p expression was strongly upregulated in DT tissues while reduced in colorectal cancer (CRC) tissues. Moreover, the miR-143-3p expression in DT tissues is consistent with plasma levels in FAP patients. In previous studies, miR-143-3p has been identified as highly expressed in mesenchymal cells (58). Bulk levels are based on the aggregation of sources, so the plasma miR-143-3p concentration might be influenced by the production or uptake of DT tissue. Furthermore, it has been reported that the expression of miR-143-3p is related to cell proliferation (59, 60). Thus, circulating miR-143-3p might be a potential diagnostic and prognostic biomarker for DT, which requires more experiments to confirm, especially in sporadic DT.

## 4 Biomarkers in prediction of postoperative recurrence

### 4.1 Tumor tissue

#### 4.1.1 *CTNNB1* mutation

In addition to its specific role in AS, *CTNNB1* also has a predictive role in the prediction of postoperative recurrence. Domont et al. performed genetic testing on 155 frozen specimens of DT tissue to analyze whether the recurrence of DT was related to the mutation of *CTNNB1*. The results showed that regardless of the specific genotype, *CTNNB1* mutated tumors had a worse prognosis than those with wild-type *CTNNB1 (* 61). Furthermore, S45F mutation was identified as a significant risk factor for recurrence. A multicenter study found that the 5-year RFS were 45%, 91% and 66% for patients with the S45F mutation, WT and other mutations respectively (40). S45F mutation was an independent prognostic factor for patients with DT, rather than marginal status, tumor size, or disease site (40). Another retrospective report also described an association between S45F and the risk factors of relapse in pediatric patients. All of these evidences indicated that *CTNNB1* mutation might be a predictive biomarker in postoperative recurrence (42). Although many studies have shown the relationship between the S45F mutation and relapse, the retrospective nature of the current studies constitutes an inherent limitation, which requires prospective studies for validation.

The contribution of the specific mutation on DT local recurrence is uncertain. S45F mutation completely blocks ubiquitination, leading to a massive increase in β-catenin, which partly explains the higher recurrence. In addition, there exists indirect evidence that the recurrence of DT is related to autophagy and inhibited apoptosis. Apoptosis and autophagy are two forms of programmed cell death, promoting or inhibiting tumorigenesis in response to a tumor’s type and stage (62, 63). Braggio et al. observed that autophagy gene overexpression promoted resistance to sorafenib in *CTNNB1* S45F mutation *in vitro* and ex vivo (64). Previous research elucidated that the overexpression of antiapoptotic genes inhibited apoptosis induction, leading to resistance to therapeutics (65). The drug resistance of S45F mutation patients may be associated with their poor prognosis, in which apoptosis and autophagy may play a role. In addition, studies have shown that RUNX3, a transcription factor within the Wnt pathway, might be involved in caspase-3-dependent apoptosis (66) (**Figure 2**). All these suggest that apoptosis and autophagy may work in the process of *CTNNB1* mutation affecting the outcome of DT patients, which deserves more attention.

#### 4.1.2 Cyclins

Cyclin A is essential for the passage of cells through the S and G2M phases, which is usually accompanied with abnormal proliferation or tumor growth (67–69). As a downstream product, Cyclin A contributes to the regulation of cell cycle progression by the Wnt signaling pathway. Studies have shown that Cyclin A can influence the prognosis of DT patients. Santti K et al. observed that Cyclin A expression was significantly associated with decreased RFS (HR =1.9, P = 0.02) in a study enrolling 76 DT patients (70). However, there is fewer data about the association between Cyclin A and the recurrence of DT, and further investigations are needed to confirm these findings.

The cyclin D regulates pRB in the G1 phase of the cell cycle. During G1 phase, the pRB binds to transcription factors like E2F to regulate cell growth (71, 72). Researches show that cyclin D1 overexpression and CTNNB1 mutation are correlated in DT (p = 0.029; p = 0.034, respectively) (73, 74). However, recent studies revealed that excessive Cyclin D could not predict a high risk of recurrence and local progression (32, 70). A possible explanation for this contradiction is that the progression is caused by inhibited apoptosis rather than cell proliferation driven by Cyclin D. Upon recurrence of the tumor, increased expression of the anti-apoptotic protein Bcl-2, Bcl-XL, Survivin and transcription factor NF-κB was observed, but no cell proliferation occurred (75, 76). Therefore, the function of Cyclin D in DT remains to be determined. More comprehensive studies are required to evaluate and confirm the prognostic value of Cyclin D and investigate apoptosis in DT.

#### 4.1.3 COX2 and PDGFβ

As a downstream target of the Wnt pathway, COX2 is a key enzyme responsible for prostaglandin synthesis. It plays a significant role in CRC progression with angiogenesis and invasion by modulating the PDGF (77, 78). In DT, COX2 immunoreactivity is significantly higher than in hypertrophic scars and normal fibrous tissue (18). Mignemi et al. found that the COX2 expression correlated with PDGFβ expression and increased its activity (18). PDGFβ expression was observed in all DT samples (27/27) with a significant immunoreactivity compared to normal tissues (28). Matano et al. investigated that the recurrent DT had higher microvessel density compared with normal samples, indicating that angiogenesis was an essential component in tumor recurrence (79). These two molecules might be underlying biomarkers for the prediction of recurrence by participating in angiogenesis.

#### 4.1.4 Estrogen signaling related molecules

The estrogen-driven pathway participates in various physiological functions by regulating gene expression, which serves as the basis for many therapeutic interventions (80). Many studies have demonstrated an involvement of the estrogen receptor in the progression of the tumor, making it a common prognostic factor and an attractive therapeutic target (81–83). Most estrogen-related studies on DT are based on clinical observations. Females are more likely to develop DT, particularly during the fertile period. Epidemiological and clinical studies indicated that ERβ is an effective biomarker for predicting outcomes. Several studies demonstrated that DT overexpressed mainly ERβ instead of ERα, with an estimated expression rate of 54.5–90% (84–86). Santti et al. analyzed 83 consecutive DT samples immunohistochemically for ERβ, Cyclin D, and Ki67. A significant correlation was found between ERβ expression and the high risk of recurrence (HR=2.6) (32). Furthermore, several studies elucidated that targeted therapy on ERβ prolonged the RFS and reached complete response (CR) in 6-14 months (87, 88). In a meta-analysis of 168 DT patients, the complete and partial response rate was 51%, with the anti-ERβ therapy or combination with the NSAID therapy (31). Another clinical trial observed only one patient (134 patients who completed treatment at least 1 year) experienced a relapse after 10 years (89). These results indicate that ERβ has a close relationship with the recurrence of DT, but the underlying mechanism remains unclear. Potential downstream targets may provide clinical value.

#### 4.1.5 Poly ADP-ribose polymerase 1

PARP-1 is an enzyme belonging to the PARP family, and it accounts for more than 90% of the enzyme activity in its family. PARP-1 is essential for repairing DNA damage, including single-strand breaks and double-strand breaks. PARP-1 binds broken DNA to its N-terminal zinc finger structure, thereby producing poly ADP-ribose chains involved in DNA repair (90, 91). PARP-1 function in DT may be promising, as PARP-1 inhibitors have been used as chemo/radiosensitizers in Ewing sarcoma (92, 93). Bräutigam et al. investigated the mRNA levels of PARP-1, ERβ, progesterone receptor (PR) and androgen receptor (AR) in DT samples, and found only PARP-1 was related to early relapse (94). Although PARP-1 may promote DT recurrence, the detailed mechanism is unknown. Previous research elucidated that cells disassembled and underwent apoptosis, as PARP-1 was cleaved (95). PARP-1 requires NAD+ as a substrate for DNA repair, which means overexpressing PARP-1 might consume available NAD+ and create a metabolic vulnerability that can be targeted (96). The related metabolomics profiles for DT showed that 1-methylnicotinamide, involved in NAD metabolism, was highly expressed in the S45F tumor cell line (97), which indicated PARP-1 might have an impact on DT progression through oxidative metabolism. More experiments are needed to confirm these assumptions.

#### 4.1.6 Potential biomarkers through sequencing

Recently, with the continuous development of sequencing technology, researchers can efficiently distinguish differentially expressed genes, which allows screening of potential markers to assess the recurrent risk in DT. Using WES, Kohsaka et al. identified three genes for prognosis, namely *IFI6*, *CKLF* and *LGMN (* 98). IFI6 was the only statistically significant gene. Salas et al. screened out *FECH*, *STOML2* and *TRIP6* which were able to predict RFS (99). In addition to coding genes, Cavallini et al. found the dysregulation of miR-21-3pg and miR-197-3p also associated with *CTNNB1* mutation might affect the progression of DT (100). However, these molecules need to be further validated in animal models and tumor samples.

### 4.2 Peripheral blood

#### 4.2.1 Circulating tumor cells

As the precursors of tumor dissemination and metastasis, CTCs are associated with cancer metastasis and poor prognosis (101). CTCs and circulating tumor microemboli in peripheral blood have been reported as early indicators for tumor invasion (102). In contrast, the role of CTCs in mesenchymal neoplasms is poorly investigated and remains unclear. In a recent study, CTCs were identified in the peripheral blood of patients with DT after AT, especially the recurrent cases (103). This outcome looks contradictory since DT is thought to lack metastasis potential. This may be inferred from two perspectives. On the one hand, as this study focused on patients following surgery, surgical manipulation may affect the CTCs release, called intraoperative tumor metastasis. Moreover, it is also possible for CTCs to colonize their tumors of origin, a process called “tumor self-seeding” (104). CTCs could reinfiltrate and promote angiogenesis in the primary tumor, which means they can easily survive in the tumor environment from their primary organs with fewer adaptations (105). This process could have consequences for tumor growth and progression, which might be a potential biomarker for DT recurrence. Several studies have associated CTC count with survival outcomes after metastatic cancer and high-CTC counts have been reported in conjunction with poor prognosis (106, 107). In DT, in addition to clinical application assessment, baseline CTC counts which have prognostic value remain to be determined experimentally.

## 5 Perspectives and concluding remarks

High clinical variability and unclear mechanism of DT make it a clinical dilemma. Thus, it is important to find molecules as reliable predictors of recurrence and progression. The current review summarizes potential biomarkers in DT progression and prognosis, focusing on the underlying mechanisms. Among these biomarkers, *CTNNB1* mutations have been demonstrated to have promising clinical value in assessing relapse and prognosis, particularly in AS. However, the biological roles of these molecules will require more comprehensive research in the future. Increasing sample sizes and standardization are necessary for future research to determine causality and long-term effects.

## Author contributions

ZS and BL designed this study. BL drafted the manuscript. RZ, ZS, DS and SZ revised the manuscript. GH supervised this study and contributed to manuscript revision with contributions from all of the other authors. All authors contributed to the article and approved the submitted version.

## Glossary

**Table:**

ADAM	A disintegrin and metalloproteinase
AF	Aggressive fibromatosis
APC	Adenomatous polyposis coli
AS	Active surveillance
CBF-1	C-promoter binding factor 1
CfDNA	Cell free DNA
CK1α	Casein kinase 1α
CKLF	Chemokine like factor-1
C-miRNA	Circulating microRNA
COX2	Cyclooxygenase 2
CRC	Colorectal cancer
CSL	CBF-1, Suppressor of hairless, Lag
CTC	Circulating tumor cell
CTNNB1	Catenin beta 1 gene
DLL-1/3/4	Delta-like-1/3/4
DT	Desmoid tumor
Dvl	Disheveled
ERβ	Estrogen receptor β
FAP	Familial adenomatous polyposis
FECH	Ferrochelatase
GSK-3β	Glycogen synthase kinase
HES	Hairy enhancer of split
Hey	HES-related proteins
IFI6	Interferon alpha inducible protein 6
JAG1/2	Jagged1/2
LEF	Lymphoid enhancer factor
LGMN	Legumain
LRP5/6	Low-density lipoprotein receptor-related protein 5/6
MMP	Matrix metalloproteinases
NICD	Notch intracellular domain
PARP-1	Poly ADP-ribose polymerase 1
pRB	Phosphorylated retinoblastoma protein
PDGF	Platelet-derived growth factor
STOML2	Stomatin like 2
TCF	T cell factor
Treg	Regulatory T cell
TRIP6	Thyroid receptor-interacting protein 6
VEGF	Vascular endothelial growth factor
WES	Whole exome sequencing;
